# Population genomic evidence of structured and connected *Plasmodium vivax* populations under host selection in Latin America

**DOI:** 10.1002/ece3.11103

**Published:** 2024-03-24

**Authors:** Johanna Helena Kattenberg, Pieter Monsieurs, Julie De Meyer, Katlijn De Meulenaere, Erin Sauve, Thaís C. de Oliveira, Marcelo U. Ferreira, Dionicia Gamboa, Anna Rosanas‐Urgell

**Affiliations:** ^1^ Malariology Unit Institute of Tropical Medicine Antwerp Antwerp Belgium; ^2^ Department of Parasitology, Institute of Biomedical Sciences University of São Paulo São Paulo Brazil; ^3^ Global Health and Tropical Medicine, Institute of Hygiene and Tropical Medicine Nova University of Lisbon Lisbon Portugal; ^4^ Instituto de Medicina Tropical “Alexander von Humboldt” Universidad Peruana Cayetano Heredia Lima Peru; ^5^ Laboratorio de Malaria: Parásitos y Vectores, Laboratorios de Investigación y Desarrollo, Departamento de Ciencias Celulares y Moleculares, Facultad de Ciencias e Ingeniería Universidad Peruana Cayetano Heredia Lima Peru; ^6^ Present address: Integrated Molecular Plant physiology Research (IMPRES) and Plants and Ecosystems (PLECO), Department of Biology University of Antwerp Antwerp Belgium

**Keywords:** genomic epidemiology, natural selection, parasitology, phylogeography, *Plasmodium vivax*, population dynamics

## Abstract

Pathogen genomic epidemiology has the potential to provide a deep understanding of population dynamics, facilitating strategic planning of interventions, monitoring their impact, and enabling timely responses, and thereby supporting control and elimination efforts of parasitic tropical diseases. *Plasmodium vivax*, responsible for most malaria cases outside Africa, shows high genetic diversity at the population level, driven by factors like sub‐patent infections, a hidden reservoir of hypnozoites, and early transmission to mosquitoes. While Latin America has made significant progress in controlling *Plasmodium falciparum*, it faces challenges with residual *P. vivax*. To characterize genetic diversity and population structure and dynamics, we have analyzed the largest collection of *P. vivax* genomes to date, including 1474 high‐quality genomes from 31 countries across Asia, Africa, Oceania, and America. While *P. vivax* shows high genetic diversity globally, Latin American isolates form a distinctive population, which is further divided into sub‐populations and occasional clonal pockets. Genetic diversity within the continent was associated with the intensity of transmission. Population differentiation exists between Central America and the North Coast of South America, vs. the Amazon Basin, with significant gene flow within the Amazon Basin, but limited connectivity between the Northwest Coast and the Amazon Basin. Shared genomic regions in these parasite populations indicate adaptive evolution, particularly in genes related to DNA replication, RNA processing, invasion, and motility – crucial for the parasite's survival in diverse environments. Understanding these population‐level adaptations is crucial for effective control efforts, offering insights into potential mechanisms behind drug resistance, immune evasion, and transmission dynamics.

## Graphical abstract

Studying 1474 high‐quality genomes of *P. vivax*, the main cause of malaria outside Africa, reveals high global genetic diversity across 31 countries. Latin American *P. vivax* isolates form a unique population with sub‐populations and genetic adaptations linked to regional adaptation of the parasites to their hosts and different environmental challenges. Understanding these population‐level adaptations is crucial for effective control efforts, providing insights into potential mechanisms behind drug resistance, immune evasion, and transmission dynamics in the fight against parasitic tropical diseases.

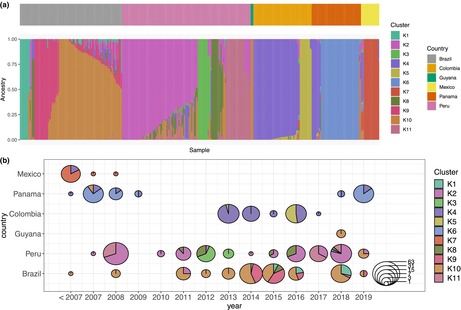

## 1 INTRODUCTION

In an era characterized by rapid environmental changes, urbanization, and increasing human‐animal interactions, the dynamics of infectious diseases are evolving at an unprecedented pace. Large‐scale programs are dedicated to controlling or eliminating infectious diseases with the greatest global health impact, with many of these efforts focused on neglected tropical diseases (NTDs). While NTDs encompass fungal, viral, and bacterial infections, the majority are caused by parasites, particularly protozoa and helminths. Vector‐borne parasitic diseases such as malaria, trypanosomiasis, leishmaniasis, and filariasis cause the greatest incidence and mortality globally (Cholewiński et al., 2015; GBD 2019 Child and Adolescent Communicable Disease Collaborators, 2023; Pearce & Tarleton, 2014).

Effective control of NTDs relies on the ability to monitor changes in pathogen populations, ensuring that interventions stay on track toward elimination goals and enabling targeted resource allocation. However, conventional monitoring techniques face challenges in many disease‐endemic countries, where diagnostic tools are often limited. This task becomes increasingly difficult as disease prevalence decreases. Genomic epidemiology, however, can provide a deep understanding of parasite population dynamics, enabling strategic planning of control interventions, monitoring their effects, and raising alerts if necessary (Kwiatkowski, 2015) and hence, support disease eradication efforts by providing actionable knowledge (Cotton et al., 2018; Gardy & Loman, 2018; Grad & Lipsitch, 2014; World Health Organization, 2022a).

While genetic data are most extensively used for diseases caused by prokaryotes and viruses (Gardy & Loman, 2018), phylodynamic tools used in viral and bacterial genomics capture both epidemiological changes and evolutionary history, due to the high mutation rates in these pathogens and measurable genetic changes within the time frame of an outbreak or epidemic (Drummond et al., 2003; Duchêne et al., 2016; Grenfell et al., 2004). However, in pathogens with a lower mutation rate and frequent recombination, such as eukaryotic parasites, inferring transmission events is more challenging (Archie et al., 2009; Prugnolle & De Meeûs, 2008). The application of genomic epidemiology for these parasitic diseases has lagged, hindered by the complexity of the parasite's life cycle and the greater size of its genome. Genetic diversity is influenced by various factors such as its life history, population dynamics, and recent changes in population size. It is crucial to have a comprehensive understanding of pathogen populations and an accurate assessment of their population structure over time to accurately evaluate the effectiveness of control interventions (Cotton et al., 2018). This information allows for a better understanding of inbreeding patterns and gene flow that can inform the development of improved strategies for controlling current populations.

While population genetics of several parasite species have been analyzed using microsatellite regions, the rapid innovation and decreasing cost of whole‐genome sequencing make it the ideal tool, since genome‐wide data have more resolution and are more comparable between populations and pathogens, eliminating the need for validated and standardized marker panels. For many key parasitic diseases, essential genomic resources like annotated reference genomes are already available. Genome‐wide data can provide insights into the sudden emergence and spread of new pathogen genotypes, reveal recent strong selection on certain genome regions, and population evolution in response to treatment and control interventions when signs of a significant bottleneck are detected. An example is the identification of emerging drug resistance in the malaria parasite *Plasmodium falciparum* (Miotto et al., 2015).

Malaria, caused by *Plasmodium* parasites, contributes to a very high disease burden with an estimated 247 million malaria cases in 84 malaria‐endemic countries (World Health Organization, 2022b). However, in several countries across the world where control efforts have reduced overall malaria cases, there has been an increase in the proportion of *Plasmodium vivax* (Price et al., 2020). Moreover, substantial reductions in *P. vivax* prevalence over 5–10 years in several locations have not consistently resulted in changes in population structure (Feachem et al., 2010; Kattenberg et al., 2020; Neafsey et al., 2012; Waltmann et al., 2018). *P. vivax* accounts for 18.0% to 71.5% of malaria cases outside Africa, with the highest proportion in the Americas, and this region contributes approximately 0.2% of global malaria cases (World Health Organization, 2022b). Venezuela, Colombia, Brazil, and Peru are the top four countries contributing the highest number of cases (79%) in the region (World Health Organization, 2022b). In contrast to other co‐endemic regions of the world, *P. falciparum* is less common except in specific regions like Colombia's Pacific coast (Rodríguez et al., 2011). Additionally, *Plasmodium malariae* infections are under‐detected in the region, despite evidence of their presence, and zoonotic transmission of *Plasmodium brasilianum* and *Plasmodium simium* between non‐human primates and humans is a concern (Recht et al., 2017). Many countries in Latin America have made strong progress in malaria control, reducing the malaria burden from 1.5 to 0.6 million cases between 2000 and 2021 (World Health Organization, 2022b). However, high transmission areas remain predominantly concentrated in the Amazon rainforest regions, disproportionally affecting indigenous and remote communities. In 2021, Venezuela, Colombia, Brazil, and Peru were in the top four countries contributing the most *P. vivax* cases (79%) in the region (World Health Organization, 2022b).

Genomic diversity in malaria parasites is generated through a combination of de novo mutations during asexual replication and sexual recombination within the mosquito vector. *Plasmodium* parasites have a high recombination rate, and frequent infections with multiple genetically distinct clones, especially in the case of *P. vivax* (Nkhoma et al., 2020; Siegel & Rayner, 2020). In addition, parasite genomes are polymorphic, with a diversity of phenotypic characteristics that impact disease severity (Neafsey et al., 2021). *P. vivax* often displays a higher genetic diversity than *P. falciparum*, due to key biological factors including frequent subpatent (i.e., detectable by molecular methods but not by field diagnostics) and asymptomatic infections, along with a hidden reservoir of hypnozoites leading to a larger number of complex infections (Olliaro et al., 2016; Sattabongkot et al., 2004). The asymptomatic infections and hypnozoites contribute to this parasite's resilience and facilitate its spread and gene flow across large regions, jeopardizing the effectiveness of local and targeted elimination strategies (Angrisano & Robinson, 2022; Auburn et al., 2021; Ferreira et al., 2022). Other factors contributing to the high genetic diversity of *P. vivax* are its longer history of association with humans, larger effective population size, and fewer population bottlenecks (Cornejo & Escalante, 2006; Hupalo et al., 2016; Neafsey et al., 2012; Noviyanti et al., 2015; Rougeron et al., 2022). Finally, sexual stages of *P. vivax* parasites appear early in the infection, facilitating effective transmission to mosquitoes before treatment, even at low‐level parasitemia, making the disease more difficult to eliminate (Bousema & Drakeley, 2011; Sattabongkot et al., 2004).

In Latin America, the analysis of mitochondrial genomes has previously shown that the combined effects of geographical population structure and the relatively low incidence of *P. vivax* malaria has resulted in patterns of low local but high regional genetic diversity (Taylor et al., 2013). In this study, we take a population genomic approach to investigate the spatial temporal dynamics of *P. vivax* in this region, using genome wide data identified through literature and supplemented with data from our own studies (*n* = 163). Using high‐resolution genome wide SNP variants (1,477,945 SNPs in the core genome) of these *P. vivax* isolates, we first compare the Latin American *P. vivax* genomes (*n* = 399) to *P. vivax* genomes from around the world (*n* = 1075). Next, we investigate the population structure, admixture, relatedness and geneflow, and signatures of positive selection to study local adaptations of the parasites. With this study, we investigate if and how the declining and more heterogenous transmission is impacting *P. vivax* population structure in this relatively recently expanded population and discuss the factors driving diversity and population structure in this ecologically diverse region. Not only is this informative for malaria control and elimination strategies, but it can also identify targets and key pathways important for *P. vivax* survival.

## 2 MATERIALS AND METHODS

### 2.1 Sequencing data

Based on an exhaustive literature search on PubMed, publications describing new *P. vivax* genomes were identified until October 2022, and the corresponding sequencing data was downloaded from the Sequencing Read Archive (SRA) of NCBI (Adam et al., 2022; Auburn et al., 2016, 2019; Benavente et al., 2021; Brashear et al., 2020; Buyon et al., 2020; Chan et al., 2012; Chen et al., 2017; Cowell et al., 2018; Daron et al., 2021; De Meulenaere et al., 2023; de Oliveira et al., 2017, 2020; Delgado‐Ratto et al., 2016; Dharia et al., 2010; Flannery et al., 2015; Hester et al., 2013; Hupalo et al., 2016; Kattenberg et al., 2022; Neafsey et al., 2012; Pearson et al., 2016; Popovici et al., 2018). Additionally, a set of new *P. vivax* genome sequencing data produced in the context of this study, were added to the list of genomes (*n* = 163, originating from Peru, Brazil, Vietnam, Eritrea, Ethiopia, Burundi, Mauritania, Somalia, and Sudan), which have been described in more detail in (De Meulenaere et al., 2022, 2023; Kattenberg et al., 2022). Briefly, DNA was extracted from leukocyte‐depleted red blood cells, whole blood or dried blood using the QIAmp DNA Blood Mini Kit (Qiagen, Germany) following the manufacturer's protocol as previously reported (De Meulenaere et al., 2022, 2023; Kattenberg et al., 2022). Parasite species was identified and quantified by a qPCR targeting Pv mtCOX1 (Gruenberg et al., 2018) using a standard curve of light microscopy quantified control isolates. Sequencing libraries were generated using Nextera XT DNA Sample Prep Kit (Illumina), or using commercial sequencing services as previously described (De Meulenaere et al., 2022, 2023; Kattenberg et al., 2022). Details of all *P. vivax* genomes used in this study can be found in Table S1, including metadata and accession numbers. The downloaded genomes contained monkey‐adapted *P. vivax* strains that were removed from population genetic analyses.

### 2.2 Ethics

Secondary use of all samples for sequencing and analysis of *P. vivax* isolates was approved through the Institutional Review Board of the Institute of Tropical Medicine Antwerp (protocols 1417/20 and 1345/19), the ethics committee at the University Hospital of Antwerp (protocol B3002020000016 and B300201523588), and Universidad Peruana Cayetano Heredia (UPCH; Lima, Peru) (protocol 101898).

### 2.3 Variant detection

Sequencing reads were first aligned using BWA version 0.7.17 to the human reference genome obtained from the Genome Reference Consortium Human Build 38 patch release 13 (GRCh38.p13). Reads not mapped in proper pairs to the human reference genome were extracted using samtools version 1.10 (flag‐F 2), and subsequently aligned to the *P. vivax* PvP01 reference genome from PlasmoDB (version 46) using BWA. Duplicate reads were removed with Picard's MarkDuplicates (version 2.22.4). Variant detection was performed using the Genome Analysis ToolKit (GATK) version 4.1.4.1, using in a first step the HaplotypeCaller command in GVCF mode for individual chromosomes. GVCF files were merged using the GenomicsDBImport, followed by genotyping using GenotypeGVCFs, resulting in one vcf file per chromosome. The vcf files were filtered according to GATK best practices: (1) SNPs were filtered out when having a QualByDepth value lower than 2, a variant quality score (QUAL) lower than 30, a StrandOddsRatio (SOR) higher than 3, a FisherStrand (FS) value higher than 60, or Root Mean Square mapping Quality (MQ) lower than 40, (2) Indels were filtered out when having a QualByDepth value lower than 2, a varia quality score lower than 30, FisherStrand (FS) value higher than 200 and ReadPosRankSum value lower than −20. Finally, for most downstream analysis the core genome (14 chromosomes, excluding subtelomeric regions and low‐complexity domains and the apicoplast and mitochondrial sequences) (Pearson et al., 2016) was selected using the BCFtools query command and samples with less than 50% of the genome covered at least 5‐fold were excluded from analysis.

### 2.4 Population structure analysis

Principal Component Analysis (PCA) was performed using PLINK software version 2.0 (Chang et al., 2015). First, only biallelic SNPs with MAF > 0.005 were selected, and linkage disequilibrium (LD) pruning was performed on the vcf file encompassing all variants in the core genome using PLINK, followed by PCA analysis using the first 20 principal components. PCA results were plotted in R using the ggplot2 library. Starting from the LD pruned dataset, admixture analysis was performed with the ADMIXTURE software version 1.3.0 (Alexander et al., 2009). The optimal number of populations was determined by running ADMIXTURE for a range of *K*‐values (i.e., number of populations) from 2 to 50. This involved a 10‐fold cross‐validation, and selection of the *K*‐value for the number of populations with the lowest cross‐validation error. Phylogenetic trees were constructed by first converting the vcf file to PHYLIP format using the vcf2phylip.py script (Ortiz, 2019). Phylogenetic trees were constructed using RAxML, with *P. knowlesi* defined as outgroup, using the GTR + G evolutionary model and using a bootstrapping value of 100 (Kozlov et al., 2019). The phylogenetic tree was visualized using the ggtree library in R. Nucleotide diversity was determined by sliding across the genome in 500‐bp windows over all LD‐pruned SNPs of the core genome using Vcftools (Danecek et al., 2011). The multiplicity of infections was calculated by estimating Wright's inbreeding co‐efficient (*F*_WS_) as a measure of the within‐host parasite diversity using the *getFws* command as implemented in the moimix package in R (Lee & Bahlo, 2016). Infections with *F*_WS_ ≥ 0.95 were considered to contain clonal (single strain) parasites, while samples with *F*_WS_ < 0.95, indicating within‐host diversity, were considered to contain multiple genetically distinct parasite strains.

### 2.5 IBD relatedness and selection analysis

Shared ancestry and relatedness between isolates was estimated using Identity‐by‐descent (IBD). PED and MAP file formats were created using VCFtools from an LD‐pruned vcf dataset of the full genome (core + (sub)telomeric and low complexity regions of the 14 chromosomes) filtered on a MAF of 0.001 based on the frequency in all included 1474 genomes. IBD‐sharing between pairs of samples, using all 399 samples from LAM, was calculated using the isoRelate package in R, which can analyze IBD in haploid recombining microorganisms in the presence of multiclonal infections (Danecek et al., 2011; Henden et al., 2018). Genetic distance was calculated using an estimated mean map unit size from *Plasmodium chabaudi* of 13.7 kb/centimorgan (cM) (Martinelli et al., 2005; Rovira‐Vallbona et al., 2021). We set the thresholds of IBD at the minimum number of SNPs (*n* = 20) and length of IBD segments (5000 bp) reported to reduce false‐positive calls using an error of 0.001. IBD has been shown to be superior to probabilistic models such as STRUCTURE for understanding the relatedness and interconnectivity of parasite populations (Henden et al., 2018; Taylor et al., 2019; Wesolowski et al., 2018). Networks of IBD‐sharing (>10% of the genome shared) between individuals were created using the igraph package in R, and the cumulative level of IBD‐sharing between isolates in countries in the network was plotted as a connection map with Scimago graphica (Hassan‐Montero et al., 2022) and used as a measure of connectivity between countries.

For the samples from Latin America, the proportion of pairs of isolates sharing IBD, as well as significance of IBD‐sharing was calculated using the isoRelate package in R for all samples together and subdivided by population, based on country, as a measure of positive selection.

### 2.6 Pathway enrichment analysis – GO terms

Gene Ontology (GO) categories were sourced from PlasmoDB release 46, with each gene being associated with one or more GO categories. To analyze a list of specific genes, a gene set enrichment analysis was conducted utilizing the hypergeometric distribution, which assesses the statistical significance of the overlap between a gene list and the assigned GO categories based on their respective counts.

## 3 RESULTS

### 3.1 *P. vivax* genomic data summary

Based on a literature search including manuscripts published before October 2022, we identified 1311 high‐quality publicly shared *P. vivax* genomes. Raw sequencing data were downloaded and all genomes were combined, including in‐house sequenced *P. vivax* genomes (*n* = 163) samples originating from Peru, Brazil, Vietnam, and imported cases in Belgium from travelers and migrants (De Meulenaere et al., 2022, 2023; Kattenberg et al., 2022).

**FIGURE 1:**
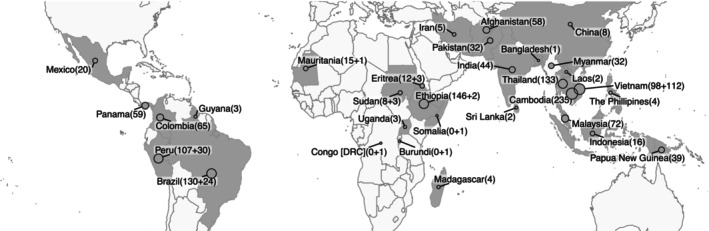
Origin of *Plasmodium vivax* genomes per country included in the analysis. Size of the dots are proportional to the number of samples in the genome dataset, and the colors indicate the country. Dots are plotted at the centre of the country (as defined by the ggmap package in R).

A total of 1474 high‐quality *P. vivax* genomes (Table S1), coming from 36 countries in Asia (*n* = 878), Americas (*n* = 399), and Africa (*n* = 197), and collected between 2000 and 2019, were retained after removing samples with less than 50% of the genome covered at least 5‐fold (Figure 1). The median sequencing coverage over the PvP01 reference genome including only retained isolates was 26‐fold (range 1–763). After alignment and variant calling, a total of 2,435,842 high quality genetic variants were identified (1,983,976 SNPs and 451,866 Indels), with a total of 1,836,935 variants in the core genome region (1,477,945 SNPs and 358,990 indels).

### 3.2 Global population structure

**FIGURE 2:**
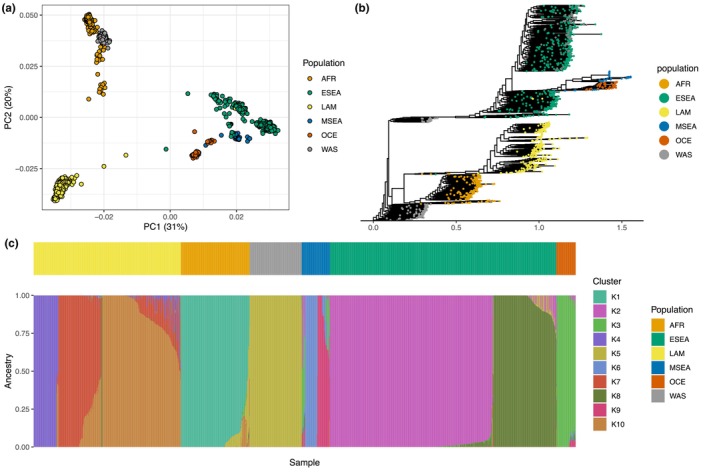
Global *Plasmodium vivax* phylogeny, admixture, and population structure. (a) Principal component analysis based on the LD‐pruned biallelic SNPs using PLINK2, showing the first two principal components. The samples (dots) are colored according to the originating population (here region). (b) Phylogenetic tree based on the LD‐pruned biallelic SNPs using RAxML, with *P. knowlesi* defined as outgroup. The phylogenetic tree was visualized without the outgroup to improve clarity of the *P. vivax* branches in the figure. (c) Admixture proportions for *K* = 10 populations using the ADMIXTURE software, with in the small bar on top the region of origin, (AFR = Africa, ESEA = Eastern South East Asia, LAM = Latin America, MSEA = Middle South East Asia, OCE = Oceania, WAS = Western Asia).

*Plasmodium vivax* genomes were grouped in regional populations (following classifications from Adam et al., 2022): Africa (AFR, including isolates from all countries in sub‐Saharan Africa, and returning travelers with history of travel to these countries), Eastern South East Asia (ESEA, including isolates from Cambodia, Laos, Thailand, Vietnam, and the China‐Myanmar border), Latin America (LAM, which includes isolates from Mexico, Central and South America), Middle South East Asia (MSEA, including isolates from Malaysia and The Philippines), Oceania (OCE, including isolates from the island of New Guinea [i.e., Papua New Guinea and part of Indonesia]), Western Asia (WAS, which includes Afghanistan, Bangladesh, India, Iran, Pakistan, and Sri Lanka). To investigate genetic clustering of *P. vivax* populations in these regions we used the biallelic SNPs as input for PCA and phylogenetic analysis. Both analyses (PCA + tree) reveal the presence of three major clusters consistent with their geographical origin (Figure 2a,b). Isolates from ESEA + MSEA form a differentiated cluster in the vicinity of isolates from OCE. Isolates from AFR cluster close to isolates from WAS, however, these two regions are clearly separated in the fourth principal component of the PCA (Figure S1) and form separate clades in the tree (Figure 2b). Isolates from LAM form a distinct cluster and clade in the PCA and tree, respectively. Together these results, with the nucleotide diversity (Figure S2), indicates a high genetic diversity within the global *P. vivax* population as a whole, with a structuring of populations by geographical region.

Admixture analysis estimated ten (*K* = 10) geographically distinct ancestral populations (Figure 2c). All genomes from AFR, WAS, and OCE were predicted to belong predominantly to a single shared ancestry within each region, while genomes from LAM, ESEA, and MSEA regions, each belong to distinct subpopulations (i.e., ancestral population within a region, Figure 2c). Admixture (predicted ancestry to more than one cluster) is mostly observed between subpopulations within a region (e.g., in LAM and ESEA), and rarely between regions, with the exception the admixture observed in AFR with WAS.

In the phylogenetic tree, isolates from WAS form two separate clades, with the upper cluster containing isolates from India (Figure 2b). This separate subpopulation could not be confirmed in the admixture analysis that estimated one ancestral cluster in this region (Figure 2c). Therefore, while Indian isolates might be distinct from other isolates in WAS, all *P. vivax* isolates from this region share a common ancestry. The highest amount of admixture between isolates is observed between the three subpopulations in LAM (mixed ancestry proportions to K7 and K10 and to a lesser extent K4), indicating a shared ancestry or gene flow between these subpopulations (Figure 2c).

### 3.3 Population structure in Central and South America

To investigate shared ancestry of *P. vivax* in Latin America at a finer geographic resolution, the population genomic analyses were repeated including only isolates from this region (*n* = 399). Results from both the PCA and the phylogenetic tree indicated clustering on a country level (Figure S3).

**FIGURE 3:**
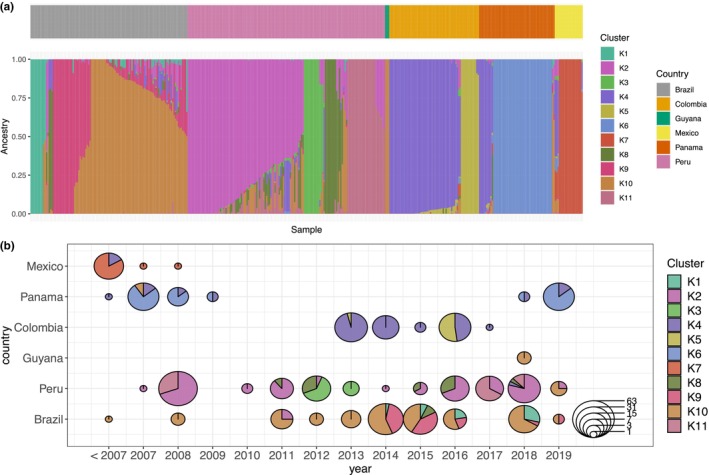
Spatio‐temporal population dynamics in Latin America. Admixture analysis of *Plasmodium vivax* samples from LAM, using *K* = 11 populations. (a) Bar plot with admixture proportions of each sample for each ancestry cluster, with in the small bar on top the country of origin for each sample. (b) Each sample is assigned to one ancestry cluster based on the highest membership probability to that population in the admixture analysis. Pie charts represent the number of samples from each cluster in that country and year.

The high degree of admixture in LAM noted in the global comparison is confirmed in this analysis and constitutes, for a large part, admixed samples within Brazil and admixture between populations from Brazil and Peru (Figure 3a). Eleven ancestral clusters (*K* = 11) within LAM were estimated (Figure 3a), and these populations are structured geographically by country or at specific locations within a country (Figure S3). In addition, admixture is observed between isolates from Colombia, Mexico, and Panama with mixed ancestry from multiple populations across LAM. Country specific ancestral populations are seen in Mexico (K7), Panama (K6), Colombia (K5), Brazil (K1 and K9), and Peru (K3 and K11). In addition, some populations are seen in multiple countries, such as isolates from Mexico and Panama that share ancestry with a population predominantly observed in Colombia (K4). While our dataset contains isolates sampled at different time periods, and populations are seen in multiple years (Figure 3b), we observed some distinct populations at specific locations, such as the Madre de Dios population (K3) in Peru, the K5 population in Tierralta in Colombia, and isolates from Manaus in Brazil (K9) (Figure S3).

Temporal analysis (Figure 3b) shows that the K10 sub‐population that is predominant in Brazil across most years, is later also observed in other countries in the Amazon Basin (2018 in Guyana, and in 2019 in Peru in a region relatively close to the border with Brazil), and in two isolates in Panama from 2007 (Figure S3). Two additional populations are seen in Brazil that are predominant in Peru (K2 and K8).

### 3.4 Gene flow across LAM

The connectivity between *P. vivax* populations in Latin American countries was assessed by measuring to what extent the parasite populations are genetically related. Pairwise IBD between all samples within and between countries was used as a measure of connectivity and parasite gene flow. From the 93,528 possible pairwise combinations of the 399 isolates from LAM, 1812 pairs of isolates (1.9%) had moderate‐to‐high relatedness (sharing 10–100% of their genome IBD). Among those, 638 pairs had high relatedness (more than 50% IBD, i.e., sibling or clonal pairs).

**FIGURE 4:**
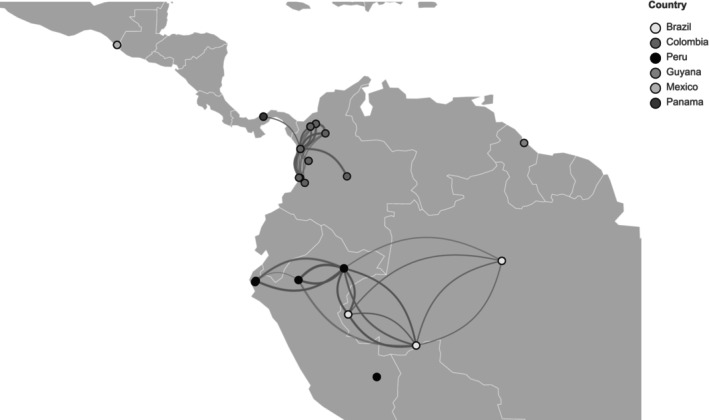
*Plasmodium vivax* IBD‐based connectivity between countries in Latin America. Connectivity network of inferred IBD between *P. vivax* samples from Latin American countries. Edges connecting countries are cumulative IBD sharing between parasite pairs with at least 10% of their genomes from those countries (numbers of samples pairs are shown in Table 1). 10% IBD‐sharing means that for these parasites at least 10% of their genomes descended from a common ancestor without intervening recombination, indicating distant to close relatedness. Node colors indicate the country of origin of the *P. vivax* genomes, and nodes were plotted on the map with known latitude and longitude of collection sites by district or if unknown in the respective country's capital (for example, in Guyana).

As expected, the majority of the related pairs (sharing 10–100% of their genome IBD) were observed within country (Figure 4 and Table 1), with observed relatedness between the different ancestral populations previously identified in Brazil (K1, K9, and K10) and Peru (K2, K8, K11) (Figure S4).

**TABLE 1:** Amount of *Plasmodium vivax* sample pairs with IBD (at ≥10% or ≥50% IBD) in pairwise analysis within and between samples from Latin American countries.

Country	Samples	Nr pairs with >50% IBD	Nr pairs with >10% IBD	Nr of possible pairs	% pairs with >50% IBD	% pairs with >10% IBD	Study references
Brazil	125	174	785	7750	2	10	Adam et al. (2022); Chan et al. (2012); De Meulenaere et al. (2023); de Oliveira et al. (2017, 2020); Hupalo et al. (2016); Kattenberg et al. (2022); Neafsey et al. (2012); Pearson et al. (2016); Benavente et al. (2021)
Colombia	65	108	149	2080	5	7	Adam et al. (2022); Hupalo et al. (2016)
Guyana	3	3	3	3	100	100	Benavente et al. (2021)
Mexico	20	41	125	190	22	66	Hupalo et al. (2016)
Panama	58	123	123	1653	7	7	Hupalo et al. (2016); Buyon et al. (2020)
Peru	162	188	591	13,041	1	5	Adam et al. (2022); Cowell et al. (2018); De Meulenaere et al. (2023); Delgado‐Ratto et al. (2016); Dharia et al. (2010); Flannery et al. (2015); Hupalo et al. (2016); Kattenberg et al. (2022)
Comparison between countries							
Brazil‐Peru	287	1	31	10,125	0.01	0.31	
Colombia‐Panama	123	0	5	1885	0.00	0.27	

A small number of related pairs (sharing 10–50% of their genome IBD) was also observed between countries: between Brazil and Peru (K10‐K2) and between Colombia and Panama (K4 and K5; Figure 4). This indicates connectivity and gene flow between the different ancestral clusters within and between countries in LAM.

To simplify subsequent analysis, parasites from Guyana are grouped with Brazil due to the low number of samples (*n* = 3) and assignment to the same population as samples from Brazil in the admixture analysis (Figure 3).

### 3.5 Diversity

Genetic diversity of malaria parasites and frequency of multiple clone infections are often associated with transmission rates, as a result of changes in the *effective population size*. We indeed observe that the genetic diversity of *P. vivax* populations in LAM (mean nucleotide diversity (pi) across the genome of 0.00071 ± 0.00046) is significantly lower (*p* < .0001) than in most other regions of the world (except MSEA, which includes mostly highly related samples from Malaysia; Figure S4). Interestingly, several clonal *P. vivax* clusters (IBD‐sharing ≥ 99% of the genome) are also observed within the LAM countries (Figure S5).

**FIGURE 5:**
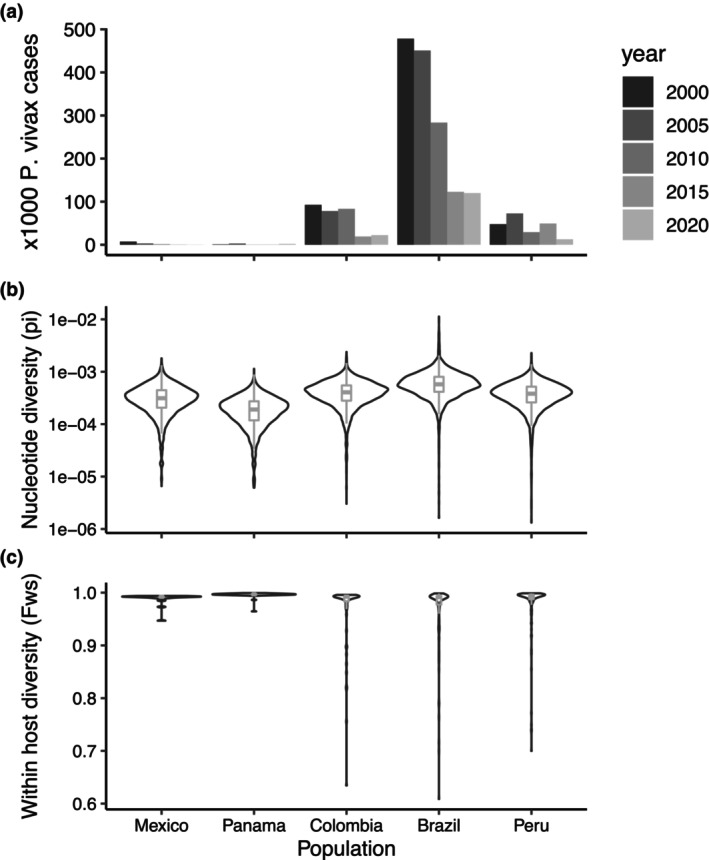
Molecular markers for transmission intensity. From the studied countries, the number of *Plasmodium vivax* cases (a) has been highest in Brazil in all years (2010–2021), data extracted from the World Malaria report 2022 (World Health Organization, 2022b). (b) Violin plot of nucleotide diversity (pi) measured across the genome in 5000 bp windows. (c) Violin plot of within‐host infection complexity assessed using within sample *F* statistic (*F*_WS_). *F*_WS_ ≥ 0.95 was considered a proxy for a monoclonal infection.

Genetic diversity was significantly different in all pairwise comparisons between LAM countries (pairwise *t*‐tests with no assumption of equal variances, *p* < .0001, Figure 5b). From the LAM countries where genomes were available for this study, Brazil has the highest number of cases, which is reflected in the diversity, that was highest in Brazil, and lowest in Panama (Figure 5a,b). While currently the prevalence of malaria in Mexico is very low, observed pi was higher than in Panama (Figure 5b). However, these samples were collected before 2007, with the majority from 2002, when reported incidence in Mexico was higher than in Panama during the years 2007–2009. Therefore, results conform with the pattern of lower genetic diversity observed at lower transmission levels. The majority of samples from LAM had monoclonal *P. vivax* infections (Figure 5c), but in the countries with higher numbers of cases (Colombia, Brazil, Peru), polyclonal infections (*F*_WS_ < 0.95) were more frequent (15.6%, 19.0%, and 10.0% of samples, respectively).

### 3.6 Regions of the genome under positive selection

To investigate positive selection in the *P. vivax* populations by country, the genome regions where isolates are identical (referred to as IBD‐segments) was determined by scanning for IBD in 5000 bp windows. Shared regions of the genome with a high amount of IBD within these populations can be indicative of positive selection.

**FIGURE 6:**
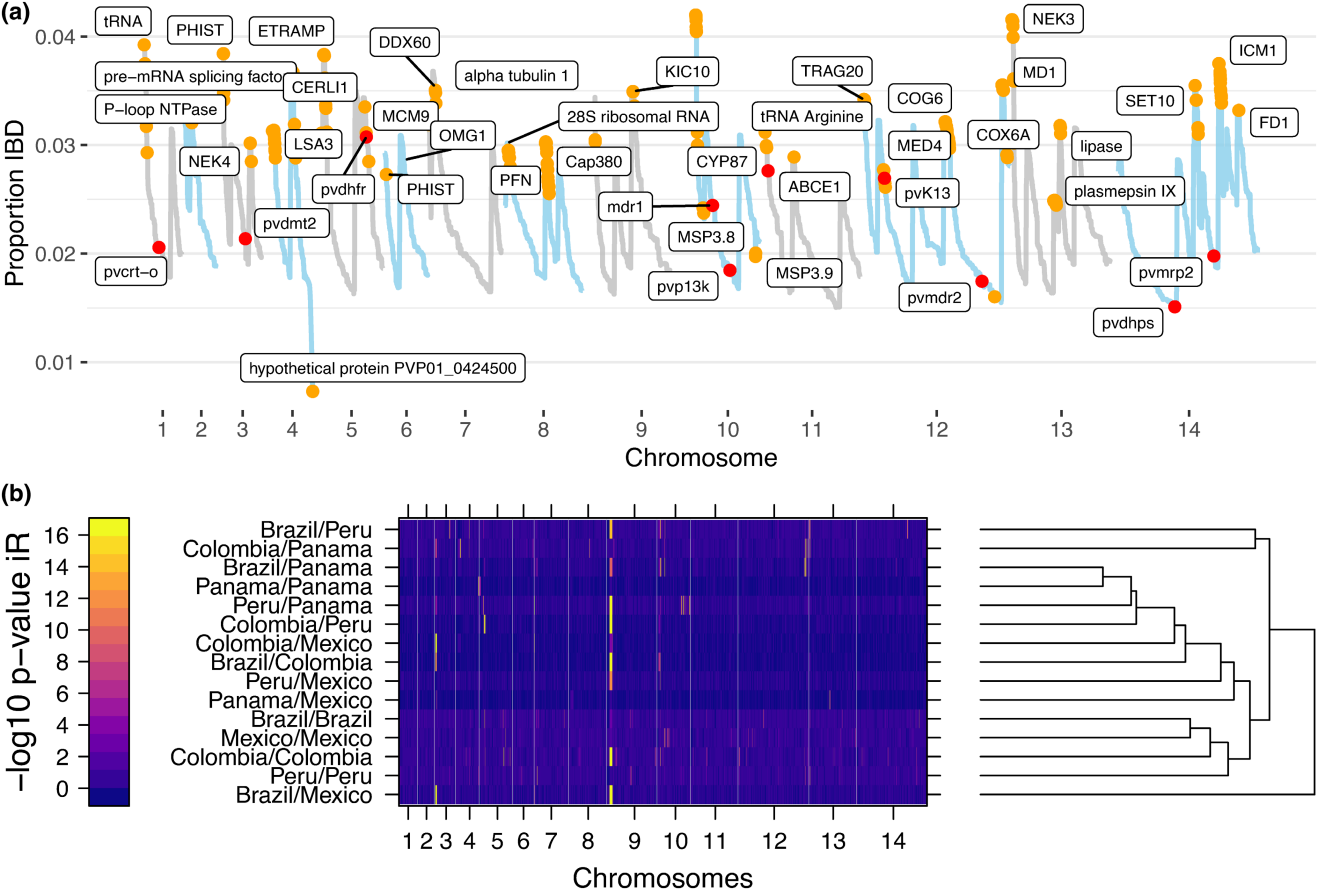
Pairwise IBD between isolates across the five populations in LAM. (a) Line plot of median IBD shared between pairs of *Plasmodium vivax* samples along the chromosomes. IBD segments with highly significant IBD are indicated in orange and annotated genes of interest at peaks of IBD‐sharing are labeled. The top genes that share significant IBD in the populations are listed in Table S2. Labeled dots in red indicate the positions and level of IBD sharing of putative drug resistant associated genes (list from Kattenberg et al., 2022). (b) Heatmap of significant pairwise IBD between populations in LAM are clustered on rows for similar patterns between populations. Low to high −log_10_ *p*‐values indicating significance levels of IBD sharing are color graded from blue to yellow. Significant IBD‐sharing is seen at a −log_10_ *p* > 1.3 (i.e., *p* < .05), and a threshold of −log_10_ *p* > 10 was used to identify highly significant areas of IBD‐sharing.

Some regions were very similar in many isolates (high proportion of IBD‐sharing), while other regions showed more variety between isolates (low proportion of IBD sharing; Figure 6a). The IBD‐segments that are shared by the greatest number of isolates are shared by a maximum of 4% of isolates (Figure 6). While this is a relatively low proportion, this is as expected from an admixed and recombining parasite population.

The highest amount of IBD‐sharing was observed near and in sub‐telomeric regions of the chromosomes, rather than in the core genome. Since IsoRelate can be sensitive to local differences in SNP density, and difficulties in accurate read mapping and variant calling in these regions can increase SNP density, we investigated whether the signals overlapped only in high SNP density regions (Figure S6). However, high IBD‐sharing was also seen in regions where SNP density was similar this was caused by a higher SNP density (Figure S7). High Tajima's D‐values were also observed near the subtelomeric regions, confirming the selection signals (Figure S8).

The genes in regions with the highest amount of IBD‐sharing between isolates (peaks in Figure 6a) in the populations are listed in Table S2. This includes, for example, two peaks on chromosome 4 (Figure 6a) that contain putative genes liver stage antigen 3 (lsa3) on the first peak and Cytosolically Exposed Rhoptry Leaflet Interacting protein 1 (cerli1) on the second peak, as well as a region with very little IBD‐sharing (i.e., valley in Figure 6a, in a genomic region with high genetic variability) containing hypothetical protein PVP01_0424500 (Figure 6a).

Significant IBD segments were investigated for each country resulting in some regions with significant IBD‐sharing in single populations, and other regions conserved in multiple populations, such as a segment at the start of chromosome 9 (Figure 6b). Since many chromosomal regions with significant IBD segments were identified, we investigated the gene ontologies to determine which pathways were enriched in IBD segments of the different populations. Enrichment was found in pathways that are essential for (i) parasite replication, such as DNA‐replication, binding and repair, protein folding, RNA transcription and processing, (ii) transport, (iii) invasion and antigenic variants, (e.g., lsa3, cerli1, merozoite surface protein 3 [msp3], tryptophan‐rich proteins [trag6, trag7, trag20]), (iv) microtubule‐related motility, and (v) male and female development (Table S2).

No known drug resistance associated genes or orthologues of *P. falciparum* resistance associated genes are located in the highest IBD regions, although dhfr, mdr1, and pvK13 are in areas with intermediate IBD (Figure 6a). Notably, there is an orthologue of a *P. falciparum* Kelch interacting protein (kic10) in a high IBD segment on chromosome 9, but the role of this protein and Kelch proteins in *P. vivax* drug resistance is unknown.

## 4 DISCUSSION

Here, we report the genomic analysis of the largest collection (*n* = 1474) of high‐quality *P. vivax* genomes to date, originating from 31 countries across Asia, Africa, Oceania, and Americas (Figure 1). The global *P. vivax* population displays high genetic diversity compared to high diversity *P. falciparum* populations in high transmission regions (Ocholla et al., 2014; Volkman et al., 2007), and separates into three main continental populations, i.e., Eastern Asia and Oceania, Africa and West Asia, and Latin America, similar to other reports (Adam et al., 2022; Daron et al., 2021; Ibrahim et al., 2023). The geographical clustering largely matches the previously defined zoogeographical zones with similar ecological and epidemiological characteristics and *P. vivax* relapse rates (Battle et al., 2014; Macdonald, 1957). Most of the clusters identified in the admixture analysis are relatively isolated within one region, with little admixture between regions, with the exception of Africa and West Asia, as reported previously (Benavente et al., 2021). In contrast, a high degree of admixture is observed within regions, especially in South‐East Asia and in LAM. In this study we increased the number of genomes compared to previous reports, in particular from West Africa, Vietnam, Brazil and Peru, with the largest genomic analysis of Latin American isolates (*n* = 399) to date. With additional samples from South‐East Asia compared to earlier studies (Adam et al., 2022; Benavente et al., 2021; Neafsey et al., 2012), we do not find additional subpopulations in admixture analysis in this region. However, with the increased sample size in LAM, we do find an additional subpopulation in Latin America compared to earlier studies (Benavente et al., 2021; Cornejo et al., 2015). This increased resolution is confirmed in another very recent study with additional samples from Brazil (Ibrahim et al., 2023).

*P. vivax* isolates from Latin America form a distinct group within the global *P. vivax* population, characterized by structured subpopulations, sporadic clonal clusters at specific sites, and lower genetic diversity compared to other regions in the world. This analysis confirms the pattern of low‐local but relatively high‐regional genetic diversity in LAM measured previously using microsatellites (Taylor et al., 2013).

The lower genetic diversity of *P. vivax* in LAM might be explained by a founder effect of relatively recent colonization events of this parasite species in this region, potentially through multiple waves of human migrations (Rodrigues et al., 2018; Rougeron et al., 2022; Steverding, 2020; van Dorp et al., 2020). In addition, a lower transmission intensity in LAM compared to other regions in the world can contribute to the lower diversity (Neafsey et al., 2012, 2021). In turn, Brazil, which is the country with the highest incidence of malaria − among those included in the LAM region in this study – exhibited the greatest level of genetic diversity. Furthermore, in comparison to genetic diversity of *P. falciparum* populations in high transmission African regions *P. vivax* diversity in LAM is high (Ocholla et al., 2014; Volkman et al., 2007). This is typically the effect of random genetic drift in small populations that remain relatively isolated from each other; rare alleles disappear (decreased diversity) and increased divergence between sites. Gene flow and admixture were detected across populations from Mexico, Panama, and the North Coast of Colombia, and across countries in the Amazon region, however connectivity between these two separate regions is highly limited. This marked population differentiation between these two regions might be attributed to factors such as differentiated ecological environments, distinct vector populations, different human occupation and mobility patterns, and history of antimalarial interventions (Batista et al., 2015; Siqueira et al., 2016; Van den Eede et al., 2010). These differentiation patterns are very similar to those observed in *P. falciparum* populations in South America (Yalcindag et al., 2012). While the Amazon region and coastline regions of South and Central America are suitable habitats for various malaria vectors, there are ecological and human barriers between the Central and Northern South American coastlines and the Amazon region. These regions are separated by mountain ranges and have clear differences in climate and vegetation where the north coast region has a tropical climate with a dry season, while the Amazon rainforest has a tropical rainforest climate with year‐round rainfall. The observed limited connectivity of parasite populations also suggests limited human mobility and thereby limited transmission between these regions.

Indeed, the Amazon basin is a vast territory of tropical rainforest encompassing several countries and including large regions of indigenous territories intersected by numerous rivers and scattered cities and villages that offer natural barriers contributing to the observed subpopulation structure (Siqueira et al., 2016). On the other hand, connectivity and admixtures within this region in all likelihood is due to patterns of human mobility, which are closely related to occupational patterns like logging and illegal gold mining (Arisco et al., 2021; Castro et al., 2019; de Castro et al., 2006; Pacheco et al., 2019; Rosas‐Aguirre et al., 2016; Torres et al., 2022). In addition, transmission of malaria (and gene flow) across large geographic distances is facilitated by the large human reservoir of asymptomatic and low‐density *P. vivax* infections characteristic for this region (de Oliveira et al., 2020; Ferreira et al., 2022) as well as the dormant reservoir of hypnozoites characteristic for *P. vivax* in general (White et al., 2014).

Conversely, further North, in Colombia, Panama, and Mexico we observe less admixture than in the Amazon region and distinct populations by country. We identified a specific subpopulation that is predominant in Colombia, but also observed (in a lower proportion) in Panama and even into Mexico likely reflecting human migration facilitating spread of this population from the North coast of Colombia into countries further to the North. According to a recent study, the majority of *P. vivax* genomes in Panama are part of a highly clonal population that has been present in the country for at least 10 years (Buyon et al., 2020). In addition, the same study reported likely imported parasites, which we identified to belong to a Colombian population.

We identified genomic regions shared across Latin American parasite populations that exhibit signatures of positive selection. Genes in these shared regions are predominantly involved in DNA replication binding and repair, RNA transcription and processing, parasite invasion, as well as microtubule‐related motility, suggesting that genes involved in these biological processes are key for *P. vivax* evolution and survival. Positive selection on DNA replication, binding, and repair genes suggests adaptive evolution to environmental challenges such us host immune responses, while positive selection on genes associated with RNA transcription and processing hints at the potential role of post‐transcriptional gene regulation in parasite adaptation and survival to varying conditions (Parobek et al., 2016), such as parasite transition between hosts or life cycle stages (*such as the male and female development through MD1 and FD1*). Moreover, microtubules are essential components of the cytoskeleton and play a critical role in cell division, motility, and intracellular transport.

The selection signals in LAM from this study are in agreement with other studies of *P. vivax* populations from South America (Cornejo et al., 2015; Ibrahim et al., 2023), supporting the validity of our IBD‐approach including polyclonal infections for selection analysis. Considering the similar approaches and findings from these studies using partly the same data, we did not further confirm selection signals with other methods. The selection signals identified in LAM are dissimilar to *P. vivax* populations in other regions, as previous investigations with global *P. vivax* isolate collections detected selective sweeps at drug resistance‐associated loci (e.g., *dhfr*, *dhps*, and *mdr1*), (Hupalo et al., 2016; Pearson et al., 2016). However, these studies also found evidence of local adaptation within distinct *P. vivax* populations and differential selection on surface antigens such as *msp* genes. In contrast to other studies, we included sub‐telomeric regions in the selection analysis. We identified genes under selection putatively involved in antigenicity and host–parasite interactions, including parasite invasion, which is poorly understood in *P. vivax*. Positive selection in invasion genes has the potential to identify new candidate genes with a role in reticulocyte invasion and hence can inform vaccine development, for example, against antigens such as *lsa3* and *msp3* found under selection here. In *P. falciparum*, *lsa3* is a candidate vaccine target and has been used to elicit sterile immunity in animal models (Daubersies et al., 2008; Ghosn et al., 2018; Perlaza et al., 2008).

Sub‐telomeric regions are often excluded in genome analysis due to challenges in aligning their highly variable and polymorphic short reads, particularly with the incomplete PvSalI reference genome, but newer genomes like PvP01 now provide improved assembly for these regions (De Meulenaere et al., 2023; Minassian et al., 2021). Our results of positive selection in these sub‐telomeric regions match well with a proposed model of *P. vivax* adaptation from a genomic study of *P. vivax* strains compared to *P. cynomolgi* and *P. knowlesi* (Cornejo et al., 2015). That study proposed that positive and negative selection might be less effective in low recombination areas (i.e., in the core genome). They suggested that in *P. vivax*, genome structure might be an adaptive mechanism to deal with changing environments, like the host's immune system, in addition to adaptations through genetic variation. In contrast, in *P. falciparum* populations, which need to survive in the host for a longer period before they can be transmitted, strong selection signals and high IBD are found in the core genome, for example, surrounding drug resistance associated genes (Amambua‐Ngwa et al., 2019; Oyebola et al., 2017; Verity et al., 2020).

This study used a convenience sampling approach, limiting the ability to generalize findings. The majority of genomes included were from Brazil, Peru, and the Colombian coastal region, while genomes from other high *P. vivax* burden areas such as Venezuela, Nicaragua, and the Amazon region of Colombia are lacking. To complete the picture of genetic diversity in Latin America genomes from these regions, often remote or challenging to access, need to be generated.

This regional analysis of *P. vivax* populations in Latin America highlights the significant genetic diversity within the continent, and regional adaptation of the parasites to their hosts and different environmental challenges, which may contribute to the resilience of *P. vivax* to current malaria control strategies. Connectivity patterns between parasite populations are highly relevant for control and elimination programs in the region where human mobility is a major driver of malaria transmission. IBD‐analysis confirmed connectivity between different ancestral clusters within and between countries, while showing a lack of connectivity between parasites in northern regions of Central and South America and the Amazon parasite population. While temporal differences in relatedness can serve as evidence for changes in transmission over time (Arambepola et al., 2023), the populations identified were very stable over time, indicating ongoing and stable transmission.

Genomic surveillance of parasitic pathogens, coupled with epidemiological data, offers valuable insights for control and elimination programs. This study highlights the use of genetic information to uncover patterns to address key epidemiological questions related to *P. vivax* in Latin America, such as the observed association between transmission patterns and diversity in LAM, and lack of connectivity between populations in the North Coast and the Amazon Basin.

Finally, as malaria risk is increasing in spatial and temporal variability worldwide, the diversity of available malaria control tools continues to grow, and funding constraints on control programs increase, there is an increasing need for malaria‐endemic countries to adopt intervention policies that move away from a one‐size‐fits‐all approach to one that is specifically tailored to their subnational context. However, the high level of connectivity between regions and countries observed in this study rather shows that a wider view and regional approach remains very important in this continent and should remain incorporated in regional malaria control and elimination strategies.

## AUTHOR CONTRIBUTIONS

**Johanna Helena Kattenberg:** Conceptualization (equal); formal analysis (equal); methodology (lead); resources (equal); visualization (equal); writing – original draft (lead); writing – review and editing (lead). **Pieter Monsieurs:** Conceptualization (equal); data curation (lead); formal analysis (equal); methodology (equal); visualization (equal); writing – original draft (supporting); writing – review and editing (supporting). **Julie De Meyer:** Data curation (equal); formal analysis (supporting); writing – review and editing (supporting). **Katlijn De Meulenaere:** Data curation (equal); formal analysis (supporting); resources (equal); writing – review and editing (supporting). **Erin Sauve:** Data curation (supporting); resources (equal); writing – review and editing (supporting). **Thaís C. de Oliveira:** Resources (equal); writing – review and editing (supporting). **Marcelo U. Ferreira:** Resources (equal); writing – review and editing (supporting). **Dionicia Gamboa:** Resources (equal); writing – review and editing (supporting). **Anna Rosanas‐Urgell:** Conceptualization (supporting); funding acquisition (lead); methodology (supporting); project administration (lead); supervision (lead); writing – original draft (supporting); writing – review and editing (supporting).

### OPEN RESEARCH BADGES

This article has earned an Open Data badge for making publicly available the digitally‐shareable data necessary to reproduce the reported results. The data is available at https://www.ncbi.nlm.nih.gov/sra, https://www.ebi.ac.uk/ena, https://www.ebi.ac.uk/eva/, and https://github.com/pietermonsieurs/pvivax_genomes.

## Supporting information


Figure S1.

Table S1.

Table S2.

## Data Availability

Related metadata of all genomes analyzed in this study can be found in Table S1. This includes the accession numbers for all raw sequences used. Genotype data in the form of a vcf file that was used in the analysis of this study is openly available from the European Variation Archive (EVA) under project number: PRJEB65949. Scripts used for data processing, analysis and plotting are shared on github: https://github.com/pietermonsieurs/pvivax_genomes. Benefits Generated: Benefits from this research accrue from the sharing of our scripts, data and results on public databases as described above.
