# Supplementary material for: Population genomic evidence of structured and connected Plasmodium vivax populations under host selection in Latin America
Source: Ecol Evol. 2024 Mar 24;14(3):e11103. doi: 10.1002/ece3.11103 (PMC10961478; doi:10.1002/ece3.11103)
Supplement: Supplementary file 1 — Figure S1. [file ECE3-14-e11103-s001.docx]

**Supplemental Information for:**

**Population genomic evidence of structured and connected *Plasmodium vivax* populations under host selection in Latin America**

Johanna Helena Kattenberg, Pieter Monsieurs, Julie De Meyer, Katlijn De Meulenaere, Erin Sauve, Thaís C. de Oliveira, Marcelo Urbano Ferreira, Dionicia Gamboa, Anna Rosanas-Urgell

**Table of Contents:**

| Supplementary figure 1 | Page 2 |
| --- | --- |
| Supplementary figure 2 | Page 3 |
| Supplementary figure 3 | Page 4 |
| Supplementary figure 4 | Page 5 |
| Supplementary figure 5 | Page 6 |
| Supplementary figure 6 | Page 7 |
| Supplementary figure 7 | Page 8 |
| Supplementary figure 8 | Page 9 |
| Description of supplementary tables | Page 10 |

Supplementary figure 1: Principal Component analysis based on the LD-pruned biallelic SNPs using PLINK2, showing the third and fourth principal component. The samples (dots) are colored according to the originating population (regions in this case).


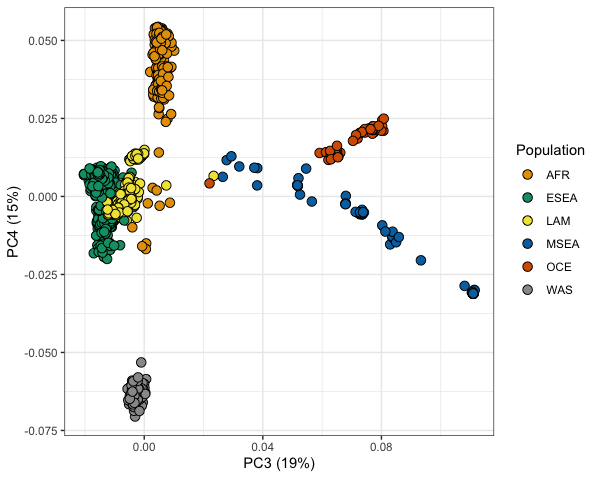


Supplementary figure 2 – Genetic diversity by regional population. Violin plot of nucleotide diversity (*pi*) measured across the complete genome in 5000 bp windows summarized by region with boxplots inset indicating median *diversity* in the population. (AFR = Africa, ESEA= Eastern South East Asia, LAM=Latin America, MSEA= Middle South East Asia, OCE= Oceania, WAS= Western Asia).


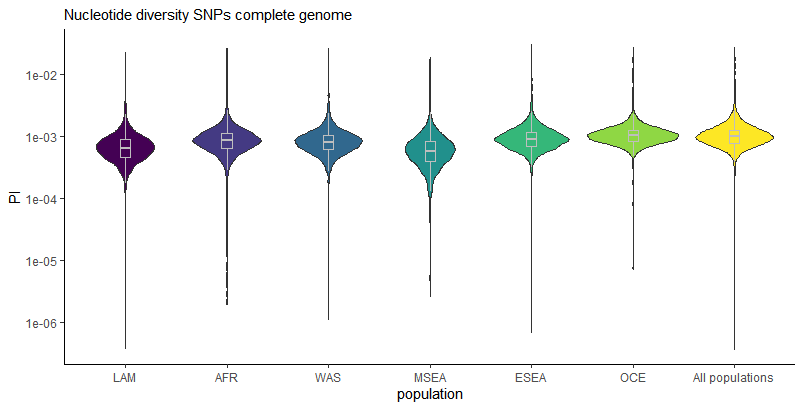


**Supplementary figure 3**: ***P. vivax* phylogeny and population structure in South America.** A) Principal Component analysis based on the LD-pruned biallelic SNPs using PLINK2, showing the first and second principal components. The samples (dots) are colored according to the originating country. B) Phylogenetic tree based on the LD- pruned biallelic SNPs using RAxML. C) Geographical mapping of the admixture proportions for K=11 populations using the ADMIXTURE software, where a pie chart indicates the distribution of the different populations for each sampling point.


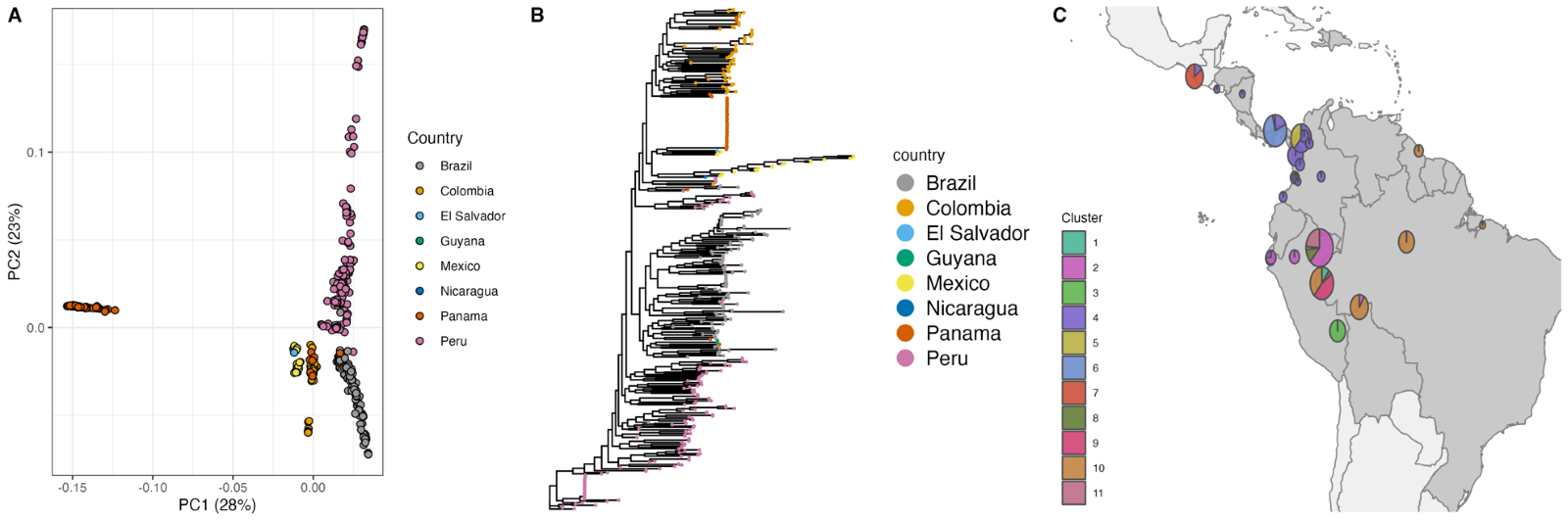


Supplementary figure 4 - **Connectivity network of inferred IBD between *P. vivax* samples from Latin American countries**. Edges connecting parasite pairs indicate that at least 10% of their genomes descended from a common ancestor without intervening recombination, indicating distant to close relatedness. Each sample was previously assigned to one ancestry cluster based on the highest membership probability to that population in the admixture analysis. Parasites from LAM that do not share >=10% IBD with at least one other parasite are not plotted. Node colors in the top figure (A) indicate country, and in the bottom figure (B) indicate the ancestral population for each sample.


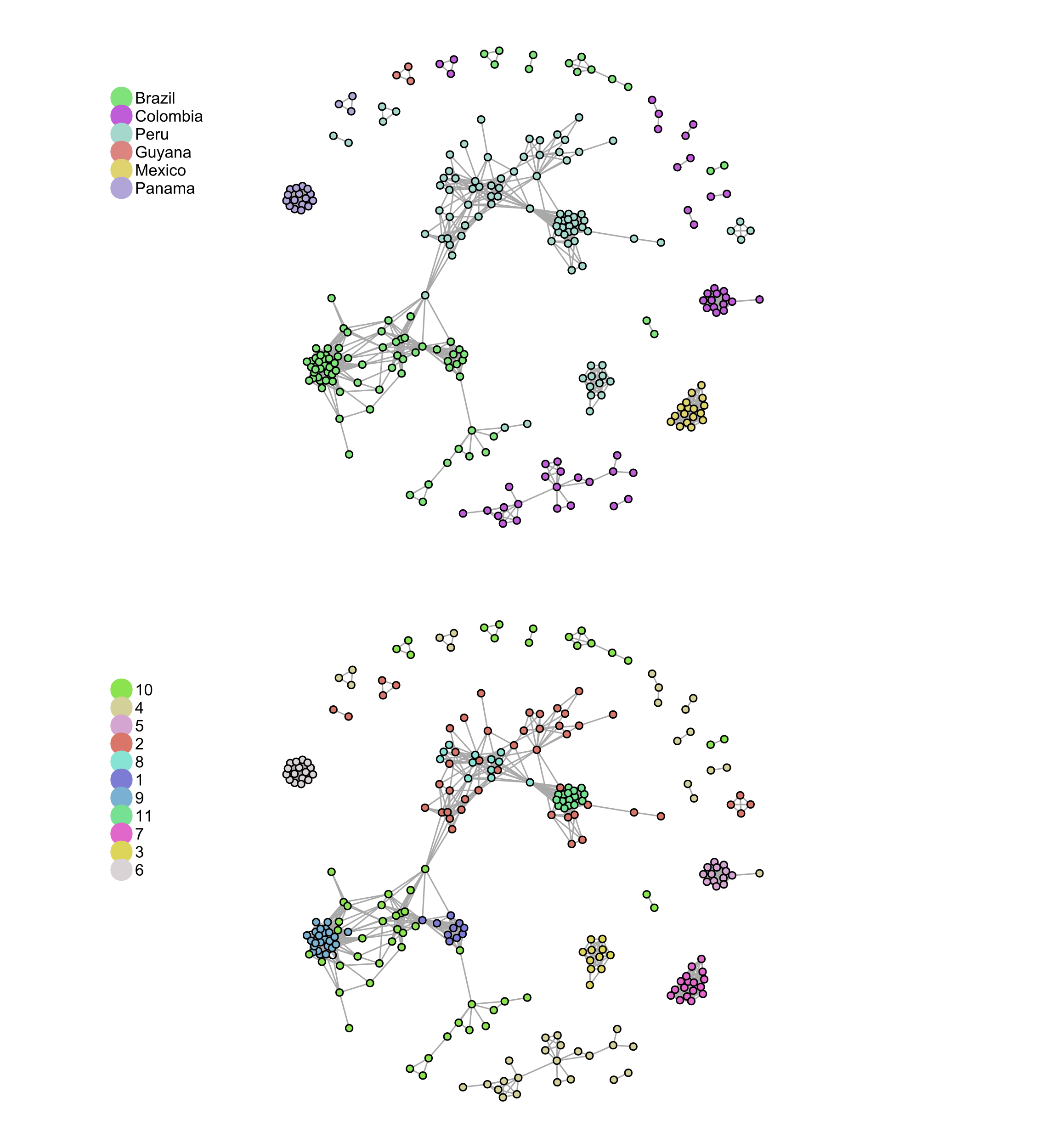


**A**

**B**

Supplementary figure 5 - **Relatedness network of inferred IBD between *P. vivax* samples from Latin American countries**. Edges connecting parasite pairs indicate that at least 99% of their genomes descended from a common ancestor without intervening recombination, indicating nearly identical parasites. Parasites from LAM that do not share >=99% IBD with at least one other parasite are not plotted. Node colors indicate the country.


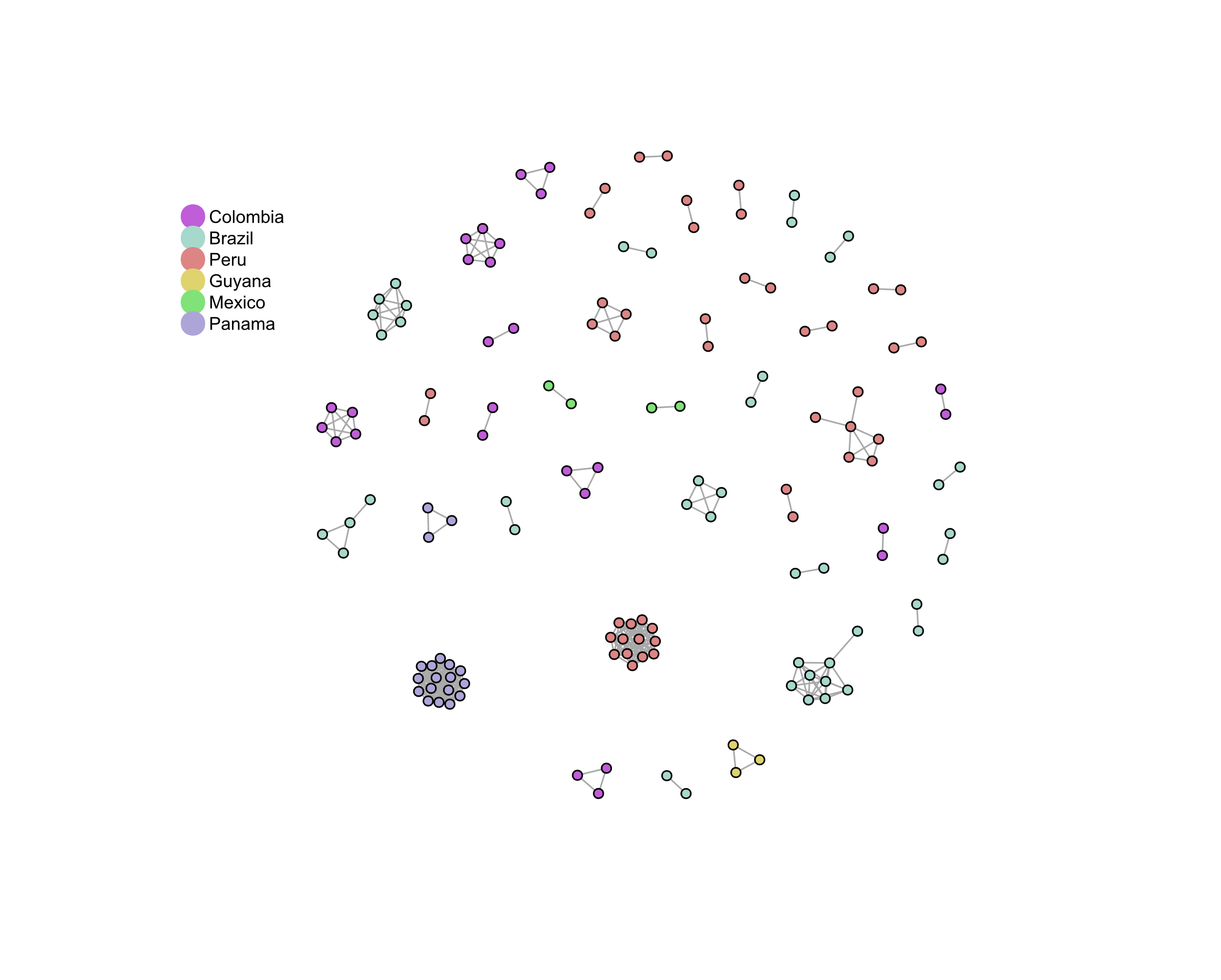


Supplementary figure 6 - Pairwise IBD between samples across the 5 populations in LAM compared to SNP density. (A) Manhattan plot of median IBD shared between pairs of P. vivax samples measured in 5000bp windows along the chromosomes. (B) SNP density (SNPs per 1000bp) estimated in 5000 bp bins across the genome. (C) -log10 p-values of pairwise IBD between populations in LAM. Significant IBD-sharing is seen at a -log10 p-value greater than 1.3 (i.e. p<0.05), and a threshold of -log10 p-value >10 was used to identify highly significant areas of IBD-sharing.​


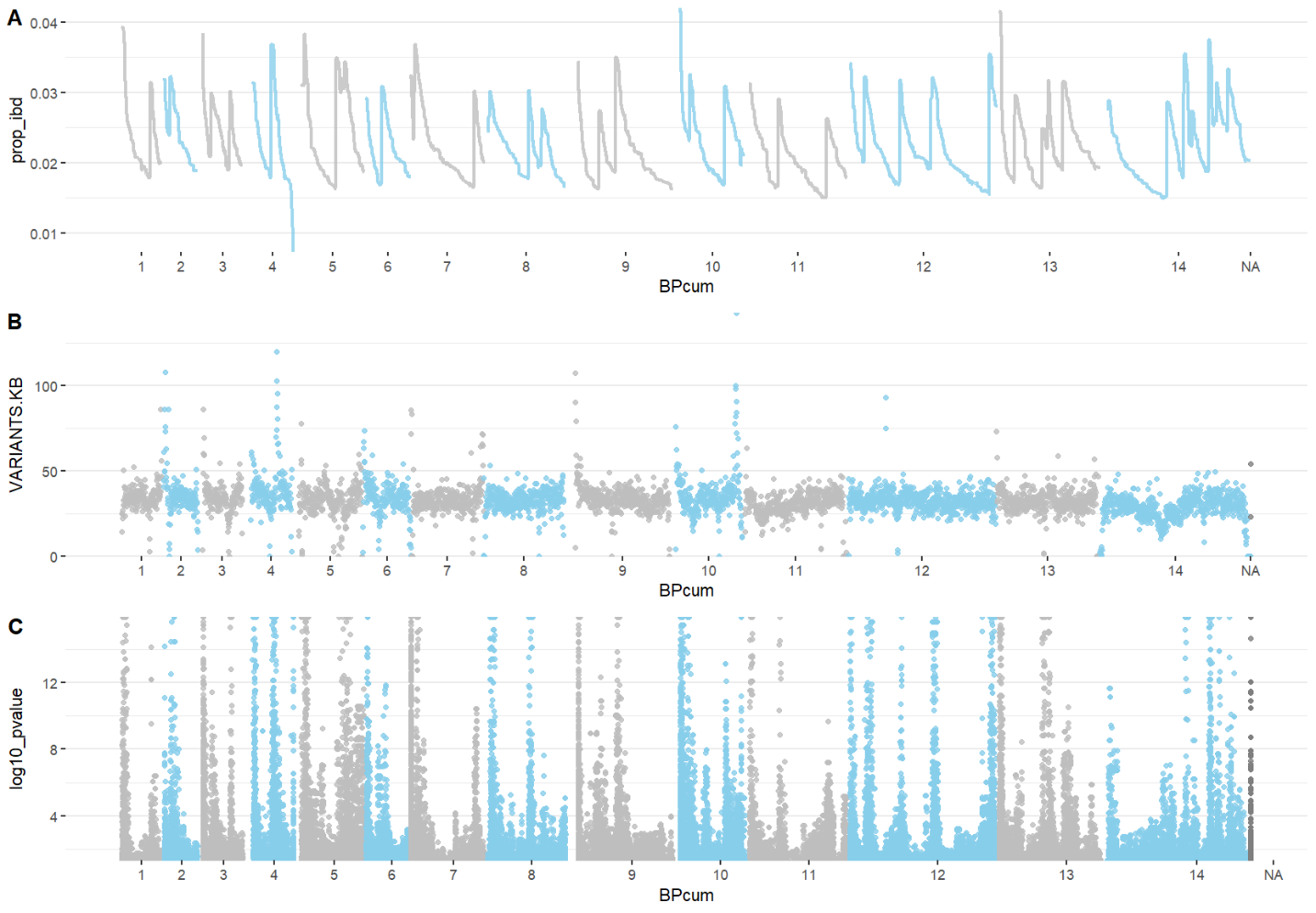


Supplementary figure 7 - Pairwise IBD between samples across the 5 populations in LAM compared to SNP density in areas where the SNP density is below 50 SNP/kbp. (A) The -log10 p-values of pairwise IBD between populations in LAM. Significant IBD-sharing is seen at a -log10 p-value greater than 1.3 (i.e. p<0.05), and a threshold of -log10 p-value >10 was used to identify highly significant areas of IBD-sharing.​ The -log10 p-values in regions of the genome where SNP density was greater than 50 SNP/kbp were removed. (B) SNP density (SNPs per 1000bp) estimated in 5000 bp bins across the genome. Regions of the genome where SNP density was greater than 50 SNP/kbp were removed.

**
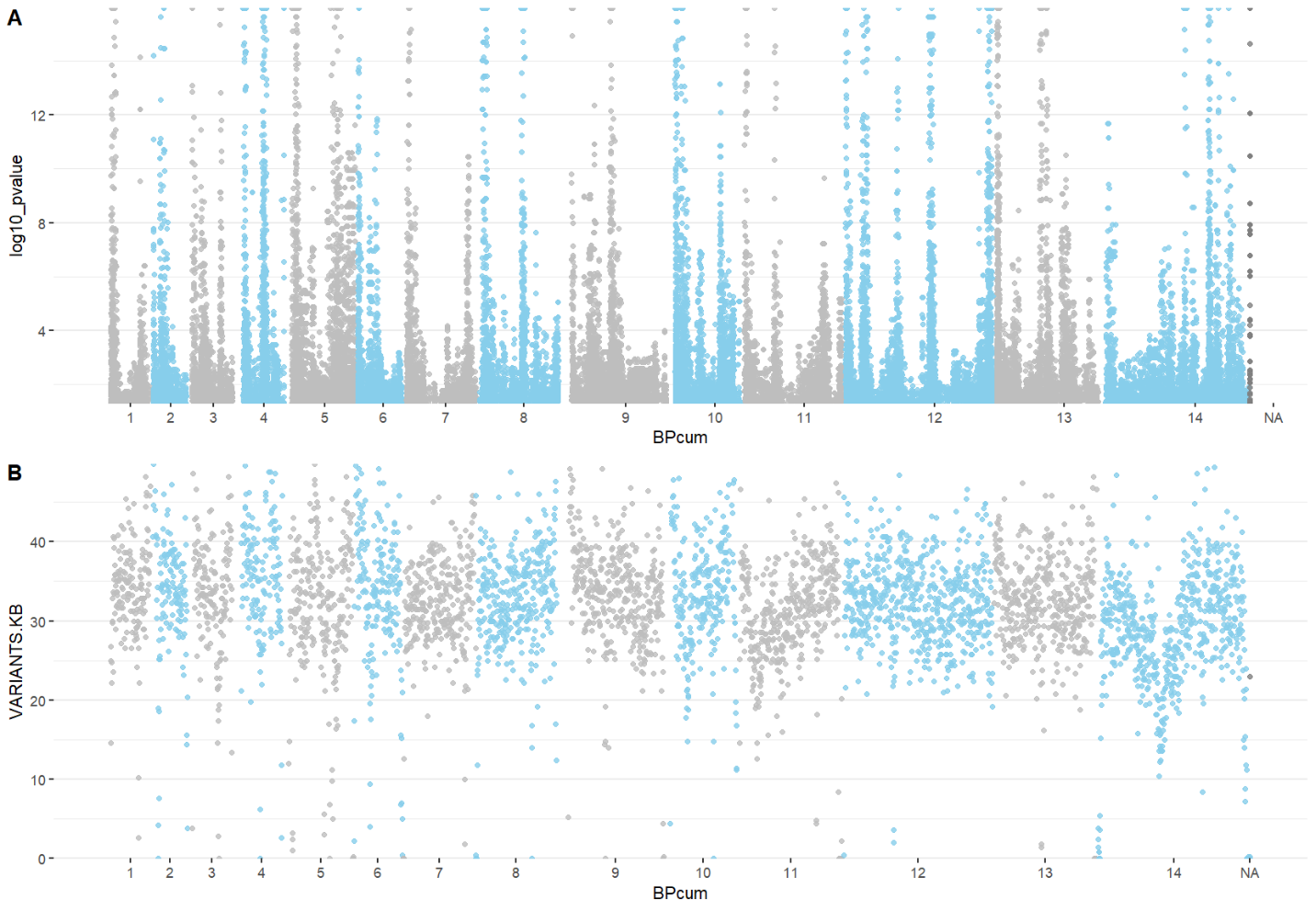
**

Supplementary figure 8 - Tajima D values across the genome determined in 5000bp windows for each population in LAM (A) Brazil (B) Colombia (C) Mexico (D) Panama (E) Peru (F) Latin America

**
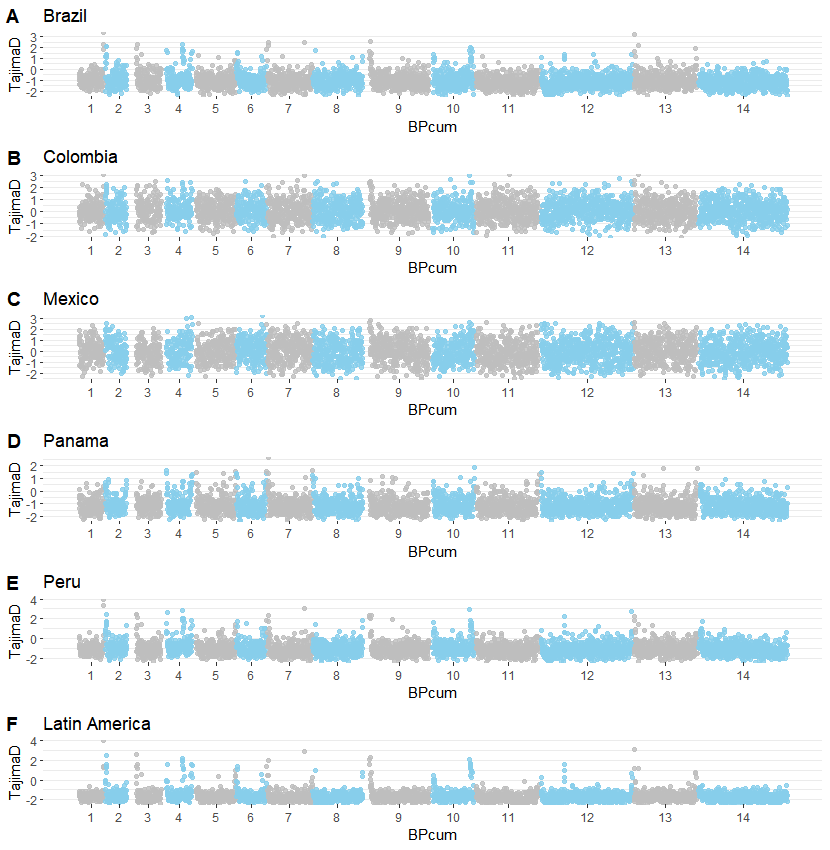
**

**Description of supplementary tables:**

Supplementary table 1: PvGenomes_master.xlsx

Excel file containing the details on the 1474 *P. vivax* genomes used in this study:

1. tab Overview_genomes: for each genome the following columns are given: Country, Location (i.e. the region within the country, if specified), Continent (i.e. the continent of origin), the population (AFR: Africa, ESEA: East South-East Asia, LAM: South and Central America, MSEA: Middle South-East Asia, OCE: Oceania, WAS: West Asia), the year of collection, the source (i.e. the scientific publication where the samples were described the first time), the SRA run accession number, the SRA BioSample accession number, ID_in_vcf (i.e. the corresponding ID in the vcf-file), perc_coverage_min_5x (i.e. the percentage of the genome covered at least by 5 reads), Fws (i.e., paramater describing the probability of multiple infections, where a value < 0.95 is indicating the presence of a multiple infection).
2. Tab genomes_per_country: the number of genomes per country in the dataset
3. Tab details_per_study: the DOI link referring to the source-column in the tab “Overview_genomes”, as well as the number of genomes retrieved form this study.

Supplementary table 2: Supplementary_table2.xlsx

Excel file containing the top genes that share significant IBD segments in the populations
